# Targeting HSP90 for cancer therapy

**DOI:** 10.1038/sj.bjc.6605066

**Published:** 2009-04-28

**Authors:** D Mahalingam, R Swords, J S Carew, S T Nawrocki, K Bhalla, F J Giles

**Affiliations:** 1 Institute for Drug Development, Cancer Research and Therapy Centre at the University of Texas Health Science Centre, San Antonio, TX, USA; 2 MGC Cancer Centre, Medical College of Georgia, Augusta, GA, USA

**Keywords:** heat-shock proteins, EGFR, HER-2, AKT, BCR-ABL, VEGF, tumour suppressor genes

## Abstract

Heat-shock proteins (HSPs) are molecular chaperones that regulate protein folding to ensure correct conformation and translocation and to avoid protein aggregation. Heat-shock proteins are increased in many solid tumours and haematological malignancies. Many oncogenic proteins responsible for the transformation of cells to cancerous forms are client proteins of HSP90. Targeting HSP90 with chemical inhibitors would degrade these oncogenic proteins, and thus serve as useful anticancer agents. This review provides an overview of the HSP chaperone machinery and the structure and function of HSP90. We also highlight the key oncogenic proteins that are regulated by HSP90 and describe how inhibition of HSP90 could alter the activity of multiple signalling proteins, receptors and transcriptional factors implicated in carcinogenesis.

Heat-shock proteins (HSPs) are a highly conserved family of molecular chaperones, some of which are induced by sublethal cellular stresses, including temperature elevation, hypoxia and oxidative damage (Young *et al*, 2004). The transcription of inducible HSPs is regulated by heat-shock factors (HSFs) in particular HSF1 (Xiao *et al*, 1999). Once present in the cell, induced HSPs provide a transient protection from subsequent, potentially lethal stress. Heat-shock proteins regulate protein folding to ensure correct conformation and translocation and to avoid aggregate formation (Young *et al*, 2004).

Heat-shock proteins are found at increased levels in many solid tumours and haematological malignancies. Their expression may in part account for the ability of malignant cells to maintain protein homoeostasis even in the hostile hypoxic and acidotic microenvironment of the tumour. Likewise, HSPs allow tumour cells to tolerate genetic alterations that would otherwise be fatal (Bagatell, 2004). Therefore, targeting of HSPs with chemical inhibitors may disrupt multiple oncogenic processes. There has thus been increased interest in the development of small molecule inhibitors that target individual HSPs to alter their function and cause the proteasomal degradation of their oncogenic ‘client’ proteins (Solit and Chiosis, 2008).

## The heat-shock response (HSR)

Chaperone proteins ensure protein homoeostasis in the presence and absence of cellular stress. In the event of protein damage, molecular chaperones facilitate protein refolding or, if the damage is irreversible, target the protein for degradation. Chaperones also participate in the post-translational modification of signalling molecules, the assembly and disassembly of transcriptional complexes and the modulation of immunogenic proteins (Bagatell, 2004).

Molecular chaperones belong to a group of highly conserved proteins called HSPs, which are classified into families according to molecular size including, HSP100, HSP90, HSP70, HSP60 and small HSP (15–30 kDa) (Young *et al*, 2004; Powers and Workman, 2007). Although the high molecular weight HSPs are adenosine triphosphate (ATP)-dependent, the small HSPs act in an ATP-independent manner (Jakob *et al*, 1993). Heat-shock proteins act in concert with co-chaperones and adaptor molecules to form large multiprotein complexes (Young *et al*, 2004; Wandinger *et al*, 2008).

The rapid induction of HSPs in response to stress is a result of a coordinated series of genetic and biochemical events referred to as the HSR. The HSR is mediated by HSFs. Of the three human HSFs, HSF1, 2 and 4, HSF1 is most crucial to the HSR (Xiao *et al*, 1999; Powers and Workman, 2007). In response to stress, HSF1 is activated by dissociation from its complex with HSP90 and HSP70. Heat-shock factor 1 binding to the heat-shock element drives the expression of responsive genes. The HSP mRNA lack introns and so are efficiently transcribed in the stressed environment without a requirement for ATP-dependent post-translational modification.

Many tumours show increased levels of HSPs (Bagatell, 2004; Whitesell and Lindquist, 2005). Heat-shock proteins promote tumour cell survival, growth and metastasis, even in growth factor-deprived conditions, by allowing continued protein translation and cellular proliferation (Whitesell and Lindquist, 2005). Heat-shock factor 1 knockout mice show reduced development of tumours, and HSF1 deficiency rendered cultured cells highly refractory to transformation initiated by mutated RAS or by platelet-derived growth factor-B (PDGF-B) overexpression (Dai *et al*, 2007; Min *et al*, 2007). Similarly, HSF1 depletion decreased viability of multiple human cancer cell lines, but had no effect on normal cells, suggesting that HSF1 provides critical relief to the cellular stresses experienced by cancer cells (Dai *et al*, 2007). Evidence also suggest that HSF1 supports tumorigenesis not only by mediating the induction of HSPs, such as HSP90, but also by orchestrating a broad network of core cellular functions that include proliferation, survival, protein synthesis and energy metabolism (Birch-Machin *et al*, 2005; Dai *et al*, 2007). Therefore, non-oncogenes like HSF1, whose functions are critical to cancer cells but dispensable to normal cells, can particularly be attractive as cancer drug targets (Solimini *et al*, 2007).

Although inhibitors of HSP90 and the proteasome disrupt chaperone machinery, they can also activate the HSR (Powers and Workman, 2007). This inadvertent induction of HSPs may attenuate the beneficial effects of HSP90 inhibitors (Banerji *et al*, 2005). Studies have found that the expression of both the major constitutive heat-shock cognate 70 (HSC70) and inducible isoform (HSP72) are increased following HSP90 inhibition with depletion of both these isoforms using short interfering RNA (siRNA) before HSP90 inhibition enhances HSP90 inhibitor cytotoxicity (Gabai *et al*, 2005; Powers *et al*, 2008). Translating these preclinical studies into early clinical trials, investigators have used the induction of HSP70 as a pharmacodynamic marker of HSP90 inhibition in both tumour tissue and peripheral blood lymphocytes (PBLs; Modi *et al*, 2007b). Whether induction of HSP70 following HSP90 inhibition attenuates the cytotoxic effects of HSP90 inhibitors remains to be determined as clinical trials mature.

As expected, studies combining inhibitors of both HSP90 and the proteasome result in the accumulation of ubiquitinated misfolded oncogenic proteins and a synergistic cytotoxicty (Mimnaugh *et al*, 2004). These findings have driven the design and implementation of clinical trials in multiple myeloma, where the proteasome inhibitor *bortezomib* has already been approved for therapeutic use.

## Heat-shock protein 90 structure and function

The HSP90 is a constitutively expressed cellular protein that compromises 1–2% of the total protein load. It is a flexible homodimer whose monomers consist of three domains, an N-terminal, ATP-binding domain, a middle (M), ATP-hydrolysis-regulating domain and a C-terminal or dimerisation domain (Wandinger *et al*, 2008). The C-terminus also regulates ATPase activity and recruits co-chaperones through a conserved EEVD motif (Wandinger *et al*, 2008). Co-chaperones, containing tetratricopeptide repeat (TPR) domains, such as HOP and PPIase, and the non-TPR co-chaperones, CDC37, p23 and Aha1, play an important role in client protein maturation and modulation of ATPase activity (Pratt and Toft, 2003; Young *et al*, 2004; Wandinger *et al*, 2008). Co-chaperones also recruit specific client proteins to HSP90 and/or stabilise HSP90 in an ATP-bound state to prolong the half-life of the mature multichaperone complex (Whitesell and Lindquist, 2005). Adenosine triphosphate hydrolysis alters HSP90 structure and promotes its chaperone function (Whitesell and Lindquist, 2005).

**Figure 1:**
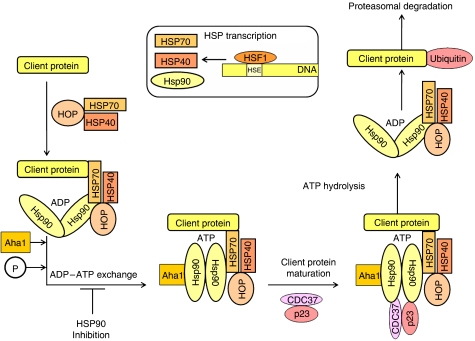
The binding of a client protein to HSP90 requires the co-operation of HSP90 with another chaperone, HSP70 and its co-factor HSP40. Both HSP90 and HSP70 chaperones are further linked by an adapter protein called HOP, which binds to both HSP90 and HSP70 through the small helical TPR domains to the C-terminal ends of HSP90 and HSP70. Aha1 is a co-factor that can bind and stimulate activity of HSP90 ATPase. When HSP90 exchanges ADP for ATP, it undergoes conformational change, which dissociates from the HSP70/HSP40/HOP complex, allowing ATP-dependent interaction with other co-chaperones, such as CDC37 and p23, to form a mature complex. It is in this mature state that HSP90 that allows client protein activation following cellular stresses, including phosphorylation of AKT, binding of EGFR to its ligands and ensuring transcription factors, such as HIF-1*α* and p53, to express genes. Inhibition of ATP-binding through HSP inhibitors prevents client protein maturation and result in degradation of these oncogenic proteins by the proteasome.

Five HSP90 isoforms have been identified to date, including cytoplasmic HSP90*α-* and *β*-isoforms, endoplasmic reticulum localised glucose regulated protein 94 (GRP94), mitochondrial tumour necrosis factor receptor-associated protein 1 (TRAP1) and membrane-associated HSP90N (Powers and Workman, 2007). All isoforms have a similar structure, except for HSP90N, which possesses a blunt N-terminus lacking an ATP-binding region (Powers and Workman, 2007). Figure 1 illustrates how HSPs regulate protein stability and maturation by formation of large multiprotein chaperone complexes.

Heat-shock protein 90 function can be further regulated by post-translational modifications, phosphorylation, nitrosylation and acetylation (Wandinger *et al*, 2008). Hyperacetylation of HSP90 occurs following histone deacetylase 6 (HDAC6) inhibition and is observed in HDAC6-deficient mouse embryonic fibroblasts and correlates with impaired HSP90 function (Zhang *et al*, 2008). Hyperacetylation attenuates HSP90 chaperoning of client proteins BCR-ABL, AKT, RAF1 and hormone receptors, such as the glucocorticoid receptor results in cellular cytotoxicity (Kovacs *et al*, 2005; Fiskus *et al*, 2007; Wandinger *et al*, 2008). These findings may explain the synergy observed between HDAC inhibitors and oncoprotein kinase inhibitors used against specific HSP90 client proteins (Fiskus *et al*, 2006).

## HSP90 and the development of cancer

Many oncogenic proteins are HSP90 client proteins. Table 1 depicts the list of client proteins discussed below, mechanism of actions of these proteins and potential tumours that HSP90 inhibitors could be applied to.

### Prosurvival signalling molecules

Growth factors drive tumour cell proliferation through transmembrane-bound receptor tyrosine kinases (RTKs), which are frequently overexpressed and/or mutated in cancer cells (Scaltriti and Baselga, 2006). The epidermal growth factor receptor (EGFR) is an RTK, which can be activated in a ligand-dependent and -independent manner or when overexpressed (Scaltriti and Baselga, 2006). Recently, several somatic mutations in the *EGFR* gene have been linked with favourable response to treatment with anti-EGFR tyrosine kinase inhibitors (TKIs), *gefitinib* and *erlotinib*, in non-small cell lung cancer (NSCLC) patients (Scaltriti and Baselga, 2006). Tumours with these mutations are also sensitive to HSP90 inhibitors despite developing resistance to TKIs. Tumour cells without *EGFR* mutations are equally resistance to both TKIs and HSP90 inhibitors (Shimamura *et al*, 2008) emphasising the importance of patient selection when designing clinical trials. Once active, EGFR is phosphorylated to initiate a series of biochemical signalling events, which diverge to activate the phosphatidylinositol 3-kinase AKT (PI3K-AKT) pathway, the Ras-Raf-extracellular signal-regulated kinase (Ras-Raf-ERK) pathway and the stress-activated protein kinase pathway. These prosurvival pathways culminate in DNA synthesis, cell division and tumour cell proliferation (Scaltriti and Baselga, 2006).

The active PI3K-AKT pathway activates prosurvival transcription factors, such as nuclear factor-*κ*B (NF-*κ*B). AKT suppress proapoptotic proteins, for example, the Forkhead transcription factor family and Bad (Jiang and Liu, 2008). Mutations in the AKT pathway, its constitutive activation or the loss of the tumour suppressor PTEN, will fuel tumour cell proliferation and prevent apoptosis (Jiang and Liu, 2008). As a HSP90 client protein, AKT degradation following HSP90 inhibition would result in attenuation of its prosurvival effects (Sato *et al*, 2000).

The Ras-Raf-ERK pathway is similarly important in cell proliferation and survival. Mutations in *N-Ras* and/or *B-Raf* constitutively activate signalling (Grbovic *et al*, 2006). Mutated B-Raf is a known HSP90 client protein. Therefore, HSP90 inhibition disrupts the mutant B-Raf and reduces ERK signalling (Grbovic *et al*, 2006). Clinical responses have been observed in patients with metastatic malignant melanoma treated with the HSP90 inhibitor *17-AAG* (Banerji *et al*, 2008) and were associated with *B-Raf* and *N-Ras* mutations, leading to a phase II trial of the HSP90 inhibitor *tanespimycin* in patients with malignant melanoma, on which clinical activity has also been observed.

ErbB2/HER-2 is another tyrosine kinase overexpressed in approximately 20–30% of breast and prostate cancers. The HER-2 protein stability is inherently dependent on HSP90. Heat-shock protein 90 inhibition decreased ErbB2 protein, phosphorylated HER-2 and downstream prosurvival signalling (Solit *et al*, 2002). Early clinical trials with *tanespimycin* in combination with the monoclonal HER-2 antibody, *traztuzumab*, showed modest clinical activity in 5 of 25 *traztuzumab* refractory patients with HER-2 overexpressing tumours (Modi *et al*, 2007b). The maximum tolerated dose was not achieved and dose escalation was terminated at 450 mg m^−2^. Following from this, the phase II trial has reported encouraging response rates in patients with HER-2-positive breast cancer, who progressed within 3 months of adjuvant *traztuzumab* therapy (Modi *et al*, 2007a). Interestingly, HSP70 induction, used as a pharmacodynamic marker of HSP90 inhibition, was observed in PBLs within 24 h of drug administration in the phase I trial (Modi *et al*, 2007b). This again raises the question as to whether the induction of HSP70 limits the clinical benefits of HSP90 inhibition.

*KIT*, another RTK, undergoes gain-of-function mutation in >90% of gastrointestinal stromal tumours (GISTs) leading to the activation of PI3K-AKT, mitogen-activated protein kinase (MAPK), STAT-1 and STAT-3 prosurvival pathways (Duensing *et al*, 2004). Inhibition of KIT oncoprotein by *imatinib mesylate* (Gleevec, Novartis, Basel, Switzerland) induces a clinical response in most GIST patients. However, many patients eventually develop resistance because of further *KIT* mutations. Salvage treatment with TKIs led to a median survival of only 15 months (Bauer *et al*, 2006). It was subsequently shown that KIT activation depended on protein stabilisation by HSP90. Heat-shock protein 90 inhibition caused degradation of both wild-type and *imatinib-*resistant KIT mutants (Fumo *et al*, 2004). Similarly, HSP90 inhibitors substantially reduced phosphorylated-KIT and total KIT expression with decreased cell survival observed in KIT-positive GIST (Bauer *et al*, 2006). This led to a phase I trial of the HSP90 inhibitor *IPI-504* in patients with refractory metastatic GIST who had progressed on TKI therapy. Although no response was seen based on RECIST criteria, FDG–PET responses based on the EORTC criteria were observed in 15 of the 18 patients (Demetri *et al*, 2006). This raises the question of what parameters of clinical response should clinical investigators look for when designing clinical trials with HSP90 inhibitors.

### Steroid hormone receptors

The oestrogen receptor-*α* (ER*α*) is an intracellular transcription factor that transduces a signal following binding of its ligand, oestradiol. Growing evidence suggests that ER*α* may be activated independent of oestradiol through the downstream signalling pathways of other activated growth factor receptors (Martin *et al*, 2000). Activated ER*α* plays a role in breast cancer by regulating genes involved in cellular proliferation (Sommer and Fuqua, 2001). In the absence of oestradiol, ER*α* is found in the nucleus as part of a multiprotein complex containing HSP90 and other chaperones (Fliss *et al*, 2000). Upon ligation, ER*α* dissociates from the complex and binds oestrogen response elements in the promoters of oestradiol-responsive genes; *progesterone receptor*, *cathepsin D* and *HDAC6* (Fliss *et al*, 2000). Oestrogen receptor-*α* may also localise at the plasma membrane to activate EGFR and insulin-like growth factor receptor and signal downstream events (Levin, 2003).

Heat-shock protein 90 inhibitors target ER*α* for proteasomal degradation (Whitesell and Lindquist, 2005), and as such may be useful in the treatment of ER-positive breast cancers. In addition, HSP90 inhibitors may be beneficial in patients who develop resistance to hormone therapies, *tamoxifen* or aromatase inhibitors (Beliakoff *et al*, 2003). These inhibitors prevent ligand-independent activation of ER*α* by preventing ER*α* phosphorylation and degrading AKT (Sato *et al*, 2000; Beliakoff *et al*, 2003).

### Chimeric fusion proteins

Chronic myeloid leukaemia (CML) is characterised by the *BCR-ABL* fusion gene, which is a constitutively active cytoplasmic tyrosine kinase that activates numerous signal transduction pathways and contributes to leukaemogenesis (Druker *et al*, 2001). *Imatinib mesylate* targets the ATP-binding site of the kinase domain of ABL and prolongs survival in all phases of CML (Druker *et al*, 2001). However, patients can develop *imatinib* resistance associated with *BCR-ABL* gene amplification and/or kinase domain mutations, which abrogate *imatinib*-binding or function (Gorre *et al*, 2001). Heat-shock protein 90 maintains BCR-ABL stability and function (Nimmanapalli *et al*, 2001). Therefore, HSP90 inhibitors targets BCR-ABL for degradation and suppresses cell proliferation (Nimmanapalli *et al*, 2001). Furthermore, HDAC inhibitors shown to effectively induce apoptosis in both *imatinib*-sensitive and -resistant BCR-ABL myeloid leukaemia cells can be used in combination with HSP90 inhibitors to synergistically induce apoptosis and inhibit cell growth (Rahmani *et al*, 2005).

Anaplastic large cell lymphomas are a subgroup of non-Hodgkins lymphoma, which are characterised by the expression of the chimeric protein, NPM-ALK. NPM-ALK originates from the fusion of nucleophosmin (NPM) and the membrane receptor, anaplastic lymphoma kinase (ALK; Hubinger *et al*, 1999). Similar to RTK, dimerisation is required for NPM-ALK activation, although, however, unlike RTKs, NPM-ALK dimers are constitutively active and do not require plasma membrane localisation (Fujimoto *et al*, 1996). Active NPM-ALK possesses phospho-transferase activity and interacts with various ALK-adapter proteins to induce cell transformation and proliferation (Hubinger *et al*, 1999). Both HSP90 and HSP70 bind NPM-ALK. Heat-shock protein 90 inhibition causes NPM-ALK destabilisation and degradation and reduces ALK-adapter protein phosphorylation. Although HSP90 inhibition prevents HSP90/NPM-ALK interaction, it results in the rapid association of HSP70 with NPM-ALK and NPM-ALK targeting for proteasomal degradation (Bonvini, 2004).

### Angiogeneic mediators

The transcription factor, hypoxia-inducible factor-1 (HIF-1), exists as a heterodimer composed of a constitutively expressed *β*-subunit and an oxygen-sensing *α-*subunit (Ebert and Bunn, 1998). Hypoxia-inducible factor-1*α* accumulates during hypoxia and dimerises with HIF-1*β* before translocating to the nucleus and binding hypoxia response elements on hypoxia-responsive genes (Ebert and Bunn, 1998). Genes regulated by HIF1-*α* are key players in cancer development and include many angiogenic mediators (*nitric oxide synthase* and *vascular endothelial growth factor* (*VEGF*)), genes involved in cellular matrix metabolism (*urokinase-type plasminogen activator receptor, matrix metalloproteinases-2 MMP2*) and glycolysis (*phosphoglycerate kinase-1* and *lactate dehydrogenase*; Weidemann and Johnson, 2008). The hypoxic microenvironment of tumours means that HIF-1*α* is often overexpressed. In normoxia, HIF-1*α* is ubiquitinated and degraded. Similarly, HSP90 inhibitors promote HIF-1*α* degradation and are more cytotoxic in hypoxic conditions (Cao *et al*, 2008).

### Apoptotic mediators

Apoptosis is a highly regulated cell death programme triggered by a diverse array of cellular stresses. Apoptotic signal transduction pathways converge to activate a conserved family of aspartic acid-specific cysteine proteases called caspases (Samali *et al*, 1999). Cells undergo apoptosis through one of the two major signalling pathways. The first, the intrinsic or mitochondrial pathway, is regulated by the Bcl-2 family proteins. Mitochondrial disturbance culminates in the release of cytochrome *c*, procaspases and other proapoptotic factors, and results in the formation of a caspase-activating complex called apoptosome. The extrinsic or death receptor-mediated pathway, is initiated by binding of an extracellular ligand (tumour necrosis factor-*α* (TNF-*α*) or TNF-related apoptosis-inducing ligand; TRAIL), formation of the death-inducing signalling complex and caspase activation (Samali *et al*, 1999).

Heat-shock proteins negatively regulate apoptosis. Heat-shock protein 90 promotes cell survival by activation of NF-*κ*B. Tumour necrosis factor-*α* activation recruits and stabilises receptor-interacting protein (RIP) at the TNF receptor-1 to maintain NF-*κ*B activity (Lewis *et al*, 2000). Key signalling molecules, such as VEGF, induces antiapoptotic protein Bcl-2 expression and stimulates HSP90 association with Bcl-2 and Apaf-1 to inhibit apoptosis (Dias *et al*, 2002). Therefore, HSP inhibitors may enhance apoptosis and circumvent resistance to anticancer treatments, such as TNF*α* and TRAIL.

### Tumour suppressor genes

As a transcription factor, p53 is activated in response to DNA-damage-inducing stresses. Once active, p53 will induce cell cycle arrest or apoptosis through its regulation of p53-responsive genes (*p21, p27, Bax, Puma, Noxa*; Vogelstein *et al*, 2000). Over 50% of human cancers possess mutated *p53* and in particular are associated with the more aggressive or chemotherapeutic resistant tumours (Vogelstein *et al*, 2000). Heat-shock protein 90 and HSP70 co-precipitate with mutant p53 (Whitesell *et al*, 1998). Heat-shock protein binding to mutant p53 inhibits mouse double minute 2-induced ubiquitination and degradation of p53. Heat-shock protein 90 inhibitors disrupt these interactions and result in p53 degradation (Whitesell *et al*, 1998). Therefore, HSP90 inhibitors could ensure that DNA-damage-inducing agents are more effective in the eradication of tumours with mutated *p53*.

### Cell cycle regulatory proteins

Cellular division is carefully monitored by cell cycle checkpoints, which are regulated by cyclins, cyclin-dependent kinases (CDK) and CDK inhibitors (Malumbres and Barbacid, 2001). In addition, retinoblastoma (Rb) and the E2F transcription factor regulate the G1/S transition. Retinoblastoma phosphorylation is crucial to cell cycle progression. When unphosphorylated, Rb blocks cell proliferation by altering E2F function. When phosphorylated, Rb is inactive and so the cell cycle proceeds (Malumbres and Barbacid, 2001). CDK4 phosphorylates Rb and is a client protein of HSP90. Heat-shock protein 90 inhibition targets CDK4 for proteasomal degradation allowing Rb to remain unphosphorylated (Srethapakdi *et al*, 2000).

Telomeres are non-coding DNA regions found at the ends of chromosomes, which contain several thousand short tandem repeats. Following a replication cycle, the telomere on each chromosome will lose 50–100 bp. This progressive shortening of telomere length eventually effects chromosome stability and leads to cell death (Xu and Neckers, 2007). In transformed cells, telomere shortening is prevented by the increased presence of telomerase, which consists of a catalytic protein component, human telomerase reverse transcriptase (hTERT) and a template-containing RNA (Xu and Neckers, 2007). Heat-shock protein 90 inhibition perturbs telomerase assembly and hTERT enzymatic activity to reduce the proliferative capacity of tumours (Xu and Neckers, 2007).

### Mediators of tissue invasion and metastasis

Tumour invasion and metastasis require the upregulation of *MMP2* and *Met* proto-oncogenes. The protein product of *Met* is the transmembrane tyrosine kinase p190^Met^, a receptor for hepatocyte growth factor/scatter factor (HGF/SF) and client protein of HSP90. Binding of HGF/SF to p190^Met^ increases cellular proliferation, migration, invasion and morphogenesis (Bottaro *et al*, 1991). Metastasis requires tumour cell adhesion to the extracellular matrix, digestion of the matrix and migration of the tumour cells. These processes are regulated by HSP90 client protein, MMP2 (Xu and Neckers, 2007). Heat-shock protein 90 inhibitors downregulate p190^Met^ expression, inhibit HGF/SF-mediated cell motility and impair MMP2 maturation (Webb *et al*, 2000).

## Conclusion

It is clear that HSP90 play a crucial role in maintaining oncogenic protein homoeostasis. Heat-shock protein 90 inhibition offers great promise in the treatment of a wide variety of solid and haematological malignancies. Various HSP90 inhibitors have entered clinical trials and have been recently reviewed (Powers and Workman, 2007; Solit and Chiosis, 2008), but many crucial questions remain: Will newer synthetic HSP90 inhibitors be more potent in cancer cells than non-transformed cells? What current pharmacodynamic indicators could be used to ascertain the optimal dosing regime? Is the induction of HSP70 in PBL a sufficiently good marker of biological activity following HSP90 inhibition? And would the induction of HSP70 amongst other HSPs decrease HSP90 inhibitor efficacy?

Emphasis has also been placed on the therapeutic targeting of other key molecules in the multichaperone protein complexes. As HSF1 regulates the expression of a number of HSPs, there has been increased interest in the development of small molecule modulators that could alter HSF1 activity and as a consequence HSP expression (Powers and Workman, 2007). Natural-acting flavonoid, quercetin; the benzylidene lactam, *KNK437*; the diterpene triepoxide triptolide and the dehydroemetine derivative NZ28 and emunin have been used to target HSF1 activity, although the specificity and potency of these compounds are still questionable (Powers and Workman, 2007). Furthermore, decreasing the normal expression levels of HSPs by HSF1 inhibition raises concerns about toxicity to non-transformed cells.

In view of HSP70 induction observed with HSP90 inhibitor treatment, the potential therapeutic benefit of modulating HSP70 activity has become attractive. As yet, there are limited small molecules inhibitors of HSP70 available; however, it would be interesting to combine HSP90 and HSP70 inhibitors that would target HSP90 and both HSC70 and HSP72 isoforms to enhance tumour response to these agents.

Early clinical trials have highlighted toxicities ranging from constitutional symptoms such as fatigue to hepatic and gastrointestinal adverse events, which have all been well tolerated (Solit and Chiosis, 2008). However, the long-term effects of HSP90 inhibition needs to be evaluated further as clinical trials mature in view of the importance of HSP90 in cellular functions and maintaining genomic stability of non-transformed cells.

In designing future clinical trials, clinical investigators will need to incorporate appropriate radiological and pathological parameters to determine the response to HSP inhibitors. This would include the use of FDG-PET and DCE-MRI to document tumour activity and avoid misinterpreted tumour responses from a CT alone. Pathological information on mutational status of *RTK* and *p53* mutations or expression levels of key signalling molecules, such as EGFR, HER-2 and HIF-1 expression, should also be obtained to select appropriate patient populations. These key signalling molecules may serve as predictive markers for HSP90 inhibitor therapy and its expression levels before and after therapy for both responders and non-responders should be documented using effective and reproducible molecular biology assays. This will help determine optimal biological dosing, preventing toxicity to normal cells and will improve efficacy of HSP90 inhibitors in various tumour types.

Novel combination therapies for each tumour type need to be developed based on preclinical data. Combinations of HSP90 inhibitors with conventional chemotherapy or targeted therapies, such as proteasomal inhibitors, HDACi, small molecule RTK inhibitors or TRAIL, may lead to greater efficacy and improved clinical outcomes.

## Figures and Tables

**Table 1:** A selected list of HSP90 client proteins, mechanism of actions of these proteins and potential target tumours that HSP90 inhibitors could be applied to

**Class of protein**	**Client protein or interacting protein of HSP90**	**Mechanism of action**	**Potential target cancer**
Receptor tyrosine kinase	EGFR mutant	Activation of downstream prosurvival pathways, such as PI3K-AKT and MAPK.	NSCLC and glioblastoma
	ErbB2/HER-2		Breast cancer
	KIT		GIST
			
Signalling molecules or kinases	AKT	Activation of prosurvival proteins and suppression of proapoptotic proteins	Various cancers
	B-Raf mutant	Constitutively activates ERK signalling	Melanoma
	MET	Involved in cellular proliferation, migration, invasion and morphogenesis	Gastric, lung
	CDK4	Phosphorylates and inactivates Rb, allowing cell cycle to proceed	Tumours with CDK4 overexpression
	Death domain kinase RIP	Allows activation of NF-*κ*B and its antiapoptotic signals	
			
Transcriptional factors	HIF-1*α*	Promoting angiogenesis	Renal cancer
	ER*α*-receptors	Regulating genes involved in cellular proliferation	Breast cancer
	P53 mutant	Transcription of genes involved in cell cycle arrest or apoptosis	Mutated in ∼50% of cancer
			
Chimeric fusion-proteins	BCR-ABL	Activates numerous signal transduction pathways in leukaemogenesis	CML
	NPM-ALK	Induces cell transformation and proliferation	Anaplastic lymphoma
			
Others	Telomerase	Prevents telomere shortening	Various cancers
	Apaf-1	Crucial for apoptosome formation	
	Bcl-2	Regulates mitochondrial apoptotic pathway	Follicular lymphoma/small cell lung cancer
	MMP2	Facilitates invasion through cell adhesion, matrix digestion and cell migration	Overexpressed in various cancers

CDK4= cyclin dependent kinase 4; CML=chronic myeloid leukaemia; EGFR=epidermal growth factor receptor; ER=oestrogen receptor; GIST=gastrointestinal stromal tumours; HIF=hypoxia-inducible factor; HSP=heat-shock protein; MAPK= mitogen activated protein kinase; NSCLC= non-small cell lung cancer; Rb=retinoblastoma; RIP=receptor-interactingprotein.

## References

[bib1] Bagatell R (2004) Altered Hsp90 function in cancer: a unique therapeutic opportunity. Mol Cancer Ther 3: 1021–1030 15299085

[bib2] Banerji U, Sain N, Sharp SY, Valenti M, Asad Y, Ruddle R, Raynaud F, Walton M, Eccles SA, Judson I, Jackman AL, Workman P (2008) An *in vitro* and *in vivo* study of the combination of the heat shock protein inhibitor 17-allylamino-17-demethoxygeldanamycin and carboplatin in human ovarian cancer models. Cancer Chemother Pharmacol 62: 769–778 18193424 10.1007/s00280-007-0662-x

[bib3] Banerji U, Walton M, Raynaud F, Grimshaw R, Kelland L, Valenti M, Judson I, Workman P (2005) Pharmacokinetic-pharmacodynamic relationships for the heat shock protein 90 molecular chaperone inhibitor 17-allylamino, 17-demethoxygeldanamycin in human ovarian cancer xenograft models. Clin Cancer Res 11: 7023–7032 16203796 10.1158/1078-0432.CCR-05-0518

[bib4] Bauer S, Yu LK, Demetri GD, Fletcher JA (2006) Heat shock protein 90 inhibition in imatinib-resistant gastrointestinal stromal tumor. Cancer Res 66: 9153–9161 16982758 10.1158/0008-5472.CAN-06-0165

[bib5] Beliakoff J, Bagatell R, Paine-Murrieta G, Taylor CW, Lykkesfeldt AE, Whitesell L (2003) Hormone-refractory breast cancer remains sensitive to the antitumor activity of heat shock protein 90 inhibitors. Clin Cancer Res 9: 4961–4971 14581371

[bib6] Birch-Machin I, Gao S, Huen D, McGirr R, White RA, Russell S (2005) Genomic analysis of heat-shock factor targets in Drosophila. Genome Biol 6: R63 15998452 10.1186/gb-2005-6-7-r63 PMC1175994

[bib7] Bonvini P (2004) Ubiquitination and proteasomal degradation of nucleophosmin-anaplastic lymphoma kinase induced by 17-allylamino-demethoxygeldanamycin: role of the co-chaperone carboxyl heat shock protein 70-interacting protein. Cancer Res 64: 3256–3264 15126367 10.1158/0008-5472.can-03-3531

[bib8] Bottaro DP, Rubin JS, Faletto DL, Chan AM, Kmiecik TE, Vande Woude GF, Aaronson SA (1991) Identification of the hepatocyte growth factor receptor as the c-met proto-oncogene product. Science (New York, NY) 251: 802–804 10.1126/science.1846706 1846706

[bib9] Cao X, Bloomston M, Zhang T, Frankel WL, Jia G, Wang B, Hall NC, Koch RM, Cheng H, Knopp MV, Sun D (2008) Synergistic antipancreatic tumor effect by simultaneously targeting hypoxic cancer cells with HSP90 inhibitor and glycolysis inhibitor. Clin Cancer Res 14: 1831–1839 18347186 10.1158/1078-0432.CCR-07-1607

[bib10] Dai C, Whitesell L, Rogers AB, Lindquist S (2007) Heat shock factor 1 is a powerful multifaceted modifier of carcinogenesis. Cell 130: 1005–1018 17889646 10.1016/j.cell.2007.07.020 PMC2586609

[bib11] Demetri GD, George S, Morgan JA, van den Abbeele A, Quigley MT, Fletcher JA, Normandt E, Patterson J, Adams J, Grayzel D (2006) Overcoming resistance to tyrosine kinase inhibitors (TKIs) through inhibition of Heat Shock Protein 90 (Hsp90) chaperone function in patients with metastatic GIST: results of a Phase I Trial of IPI-504, a water-soluble Hsp90 inhibitor. EJC Suppl 4: 173

[bib12] Dias S, Shmelkov SV, Lam G, Rafii S (2002) VEGF(165) promotes survival of leukemic cells by Hsp90-mediated induction of Bcl-2 expression and apoptosis inhibition. Blood 99: 2532–2540 11895790 10.1182/blood.v99.7.2532

[bib13] Druker BJ, Sawyers CL, Kantarjian H, Resta DJ, Reese SF, Ford JM, Capdeville R, Talpaz M (2001) Activity of a specific inhibitor of the BCR-ABL tyrosine kinase in the blast crisis of chronic myeloid leukemia and acute lymphoblastic leukemia with the Philadelphia chromosome. N Engl J Med 344: 1038–1042 11287973 10.1056/NEJM200104053441402

[bib14] Duensing A, Medeiros F, McConarty B, Joseph NE, Panigrahy D, Singer S, Fletcher CD, Demetri GD, Fletcher JA (2004) Mechanisms of oncogenic KIT signal transduction in primary gastrointestinal stromal tumors (GISTs). Oncogene 23: 3999–4006 15007386 10.1038/sj.onc.1207525

[bib15] Ebert BL, Bunn HF (1998) Regulation of transcription by hypoxia requires a multiprotein complex that includes hypoxia-inducible factor 1, an adjacent transcription factor, and p300/CREB binding protein. Mol Cell Biol 18: 4089–4096 9632793 10.1128/mcb.18.7.4089 PMC108993

[bib16] Fiskus W, Pranpat M, Bali P, Balasis M, Kumaraswamy S, Boyapalle S, Rocha K, Wu J, Giles F, Manley PW, Atadja P, Bhalla K (2006) Combined effects of novel tyrosine kinase inhibitor AMN107 and histone deacetylase inhibitor LBH589 against Bcr-Abl-expressing human leukemia cells. Blood 108: 645–652 16537804 10.1182/blood-2005-11-4639

[bib17] Fiskus W, Ren Y, Mohapatra A, Bali P, Mandawat A, Rao R, Herger B, Yang Y, Atadja P, Wu J, Bhalla K (2007) Hydroxamic acid analogue histone deacetylase inhibitors attenuate estrogen receptor-alpha levels and transcriptional activity: a result of hyperacetylation and inhibition of chaperone function of heat shock protein 90. Clin Cancer Res 13: 4882–4890 17699868 10.1158/1078-0432.CCR-06-3093

[bib18] Fliss AE, Benzeno S, Rao J, Caplan AJ (2000) Control of estrogen receptor ligand binding by Hsp90. J Steroid Biochem Mol Biol 72: 223–230 10822011 10.1016/s0960-0760(00)00037-6

[bib19] Fujimoto J, Shiota M, Iwahara T, Seki N, Satoh H, Mori S, Yamamoto T (1996) Characterization of the transforming activity of p80, a hyperphosphorylated protein in a Ki-1 lymphoma cell line with chromosomal translocation t(2;5). Proc Natl Acad Sci USA 93: 4181–4186 8633037 10.1073/pnas.93.9.4181 PMC39508

[bib20] Fumo G, Akin C, Metcalfe DD, Neckers L (2004) 17-Allylamino-17-demethoxygeldanamycin (17-AAG) is effective in down-regulating mutated, constitutively activated KIT protein in human mast cells. Blood 103: 1078–1084 14551138 10.1182/blood-2003-07-2477

[bib21] Gabai VL, Budagova KR, Sherman MY (2005) Increased expression of the major heat shock protein Hsp72 in human prostate carcinoma cells is dispensable for their viability but confers resistance to a variety of anticancer agents. Oncogene 24: 3328–3338 15735699 10.1038/sj.onc.1208495

[bib22] Gorre ME, Mohammed M, Ellwood K, Hsu N, Paquette R, Rao PN, Sawyers CL (2001) Clinical resistance to STI-571 cancer therapy caused by BCR-ABL gene mutation or amplification. Science (New York, NY) 293: 876–880 10.1126/science.1062538 11423618

[bib23] Grbovic OM, Basso AD, Sawai A, Ye Q, Friedlander P, Solit D, Rosen N (2006) V600E B-Raf requires the Hsp90 chaperone for stability and is degraded in response to Hsp90 inhibitors. Proc Natl Acad Sci USA 103: 57–62 16371460 10.1073/pnas.0609973103 PMC1325013

[bib24] Hubinger G, Scheffrahn I, Muller E, Bai R, Duyster J, Morris SW, Schrezenmeier H, Bergmann L (1999) The tyrosine kinase NPM-ALK, associated with anaplastic large cell lymphoma, binds the intracellular domain of the surface receptor CD30 but is not activated by CD30 stimulation. Exp Hematol 27: 1796–1805 10641597 10.1016/s0301-472x(99)00116-2

[bib25] Jakob U, Gaestel M, Engel K, Buchner J (1993) Small heat shock proteins are molecular chaperones. J Biol Chem 268: 1517–1520 8093612

[bib26] Jiang BH, Liu LZ (2008) PI3K/PTEN signaling in tumorigenesis and angiogenesis. Biochim Biophys Acta 1784: 150–158 17964232 10.1016/j.bbapap.2007.09.008

[bib27] Kovacs JJ, Murphy PJ, Gaillard S, Zhao X, Wu JT, Nicchitta CV, Yoshida M, Toft DO, Pratt WB, Yao TP (2005) HDAC6 regulates Hsp90 acetylation and chaperone-dependent activation of glucocorticoid receptor. Mol Cell 18: 601–607 15916966 10.1016/j.molcel.2005.04.021

[bib28] Levin ER (2003) Bidirectional signaling between the estrogen receptor and the epidermal growth factor receptor. Mol Endocrinol 17: 309–317 12554774 10.1210/me.2002-0368

[bib29] Lewis J, Devin A, Miller A, Lin Y, Rodriguez Y, Neckers L, Liu ZG (2000) Disruption of hsp90 function results in degradation of the death domain kinase, receptor-interacting protein (RIP), and blockage of tumor necrosis factor-induced nuclear factor-kappaB activation. J Biol Chem 275: 10519–10526 10744744 10.1074/jbc.275.14.10519

[bib30] Malumbres M, Barbacid M (2001) To cycle or not to cycle: a critical decision in cancer. Nat Rev Cancer 1: 222–231 11902577 10.1038/35106065

[bib31] Martin MB, Franke TF, Stoica GE, Chambon P, Katzenellenbogen BS, Stoica BA, McLemore MS, Olivo SE, Stoica A (2000) A role for Akt in mediating the estrogenic functions of epidermal growth factor and insulin-like growth factor I. Endocrinology 141: 4503–4511 11108261 10.1210/endo.141.12.7836

[bib32] Mimnaugh EG, Xu W, Vos M, Yuan X, Isaacs JS, Bisht KS, Gius D, Neckers L (2004) Simultaneous inhibition of hsp 90 and the proteasome promotes protein ubiquitination, causes endoplasmic reticulum-derived cytosolic vacuolization, and enhances antitumor activity. Mol Cancer Ther 3: 551–566 15141013

[bib33] Min JN, Huang L, Zimonjic DB, Moskophidis D, Mivechi NF (2007) Selective suppression of lymphomas by functional loss of Hsf1 in a p53-deficient mouse model for spontaneous tumors. Oncogene 26: 5086–5097 17310987 10.1038/sj.onc.1210317

[bib34] Modi S, Stopeck A, Kinden H, Sugarman S, Ma W, Solit D, Kersey K, Johnson R, Hannah AL, Hudis C (2007a) Tanespimycin (an Hsp90 inhibitor) and trastuzumab is an active combination in patients (pts) with Her2-positive trastuzumab-refractory metastatic breast cancer (MBC): phase 2 trial. Breast Cancer Res Treat 106: S269–S270

[bib35] Modi S, Stopeck AT, Gordon MS, Mendelson D, Solit DB, Bagatell R, Ma W, Wheler J, Rosen N, Norton L, Cropp GF, Johnson RG, Hannah AL, Hudis CA (2007b) Combination of trastuzumab and tanespimycin (17-AAG, KOS-953) is safe and active in trastuzumab-refractory HER-2 overexpressing breast cancer: a phase I dose-escalation study. J Clin Oncol 25: 5410–5417 18048823 10.1200/JCO.2007.11.7960

[bib36] Nimmanapalli R, O'Bryan E, Bhalla K (2001) Geldanamycin and its analogue 17-allylamino-17-demethoxygeldanamycin lowers Bcr-Abl levels and induces apoptosis and differentiation of Bcr-Abl-positive human leukemic blasts. Cancer Res 61: 1799–1804 11280726

[bib37] Powers MV, Clarke PA, Workman P (2008) Dual targeting of HSC70 and HSP72 inhibits HSP90 function and induces tumor-specific apoptosis. Cancer Cell 14: 250–262 18772114 10.1016/j.ccr.2008.08.002

[bib38] Powers MV, Workman P (2007) Inhibitors of the heat shock response: biology and pharmacology. FEBS Lett 581: 3758–3769 17559840 10.1016/j.febslet.2007.05.040

[bib39] Pratt WB, Toft DO (2003) Regulation of signaling protein function and trafficking by the hsp90/hsp70-based chaperone machinery. Exp Biol Med (Maywood, NJ) 228: 111–133 10.1177/153537020322800201 12563018

[bib40] Rahmani M, Reese E, Dai Y, Bauer C, Kramer LB, Huang M, Jove R, Dent P, Grant S (2005) Cotreatment with suberanoylanilide hydroxamic acid and 17-allylamino 17-demethoxygeldanamycin synergistically induces apoptosis in Bcr-Abl+ Cells sensitive and resistant to STI571 (imatinib mesylate) in association with down-regulation of Bcr-Abl, abrogation of signal transducer and activator of transcription 5 activity, and Bax conformational change. Mol Pharmacol 67: 1166–1176 15625278 10.1124/mol.104.007831

[bib41] Samali A, Zhivotovsky B, Jones D, Nagata S, Orrenius S (1999) Apoptosis: cell death defined by caspase activation. Cell Death Differ 6: 495–496 10381647 10.1038/sj.cdd.4400520

[bib42] Sato S, Fujita N, Tsuruo T (2000) Modulation of Akt kinase activity by binding to Hsp90. Proc Natl Acad Sci USA 97: 10832–10837 10995457 10.1073/pnas.170276797 PMC27109

[bib43] Scaltriti M, Baselga J (2006) The epidermal growth factor receptor pathway: a model for targeted therapy. Clin Cancer Res 12: 5268–5272 17000658 10.1158/1078-0432.CCR-05-1554

[bib44] Shimamura T, Li D, Ji H, Haringsma HJ, Liniker E, Borgman CL, Lowell AM, Minami Y, McNamara K, Perera SA, Zaghlul S, Thomas RK, Greulich H, Kobayashi S, Chirieac LR, Padera RF, Kubo S, Takahashi M, Tenen DG, Meyerson M, Wong KK, Shapiro GI (2008) Hsp90 inhibition suppresses mutant EGFR-T790M signaling and overcomes kinase inhibitor resistance. Cancer Res 68: 5827–5838 18632637 10.1158/0008-5472.CAN-07-5428 PMC3272303

[bib45] Solimini NL, Luo J, Elledge SJ (2007) Non-oncogene addiction and the stress phenotype of cancer cells. Cell 130: 986–988 17889643 10.1016/j.cell.2007.09.007

[bib46] Solit DB, Chiosis G (2008) Development and application of Hsp90 inhibitors. Drug Discov Today 13: 38–43 18190862 10.1016/j.drudis.2007.10.007

[bib47] Solit DB, Zheng FF, Drobnjak M, Munster PN, Higgins B, Verbel D, Heller G, Tong W, Cordon-Cardo C, Agus DB, Scher HI, Rosen N (2002) 17-Allylamino-17-demethoxygeldanamycin induces the degradation of androgen receptor and HER-2/neu and inhibits the growth of prostate cancer xenografts. Clin Cancer Res 8: 986–993 12006510

[bib48] Sommer S, Fuqua SA (2001) Estrogen receptor and breast cancer. Sem Cancer Biol 11: 339–352 10.1006/scbi.2001.0389 11562176

[bib49] Srethapakdi M, Liu F, Tavorath R, Rosen N (2000) Inhibition of Hsp90 function by ansamycins causes retinoblastoma gene product-dependent G1 arrest. Cancer Res 60: 3940–3946 10919672

[bib50] Vogelstein B, Lane D, Levine AJ (2000) Surfing the p53 network. Nature 408: 307–310 11099028 10.1038/35042675

[bib51] Wandinger SK, Richter K, Buchner J (2008) The Hsp90 chaperone machinery. J Biol Chem 283: 18473–18477 18442971 10.1074/jbc.R800007200

[bib52] Webb CP, Hose CD, Koochekpour S, Jeffers M, Oskarsson M, Sausville E, Monks A, Vande Woude GF (2000) The geldanamycins are potent inhibitors of the hepatocyte growth factor/scatter factor-met-urokinase plasminogen activator-plasmin proteolytic network. Cancer Res 60: 342–349 10667586

[bib53] Weidemann A, Johnson RS (2008) Biology of HIF-1alpha. Cell Death Differ 15: 621–627 18259201 10.1038/cdd.2008.12

[bib54] Whitesell L, Lindquist SL (2005) HSP90 and the chaperoning of cancer. Nat Rev Cancer 5: 761–772 16175177 10.1038/nrc1716

[bib55] Whitesell L, Sutphin PD, Pulcini EJ, Martinez JD, Cook PH (1998) The physical association of multiple molecular chaperone proteins with mutant p53 is altered by geldanamycin, an hsp90-binding agent. Mol Cell Biol 18: 1517–1524 9488468 10.1128/mcb.18.3.1517 PMC108866

[bib56] Xiao X, Zuo X, Davis AA, McMillan DR, Curry BB, Richardson JA, Benjamin IJ (1999) HSF1 is required for extra-embryonic development, postnatal growth and protection during inflammatory responses in mice. EMBO J 18: 5943–5952 10545106 10.1093/emboj/18.21.5943 PMC1171660

[bib57] Xu W, Neckers L (2007) Targeting the molecular chaperone heat shock protein 90 provides a multifaceted effect on diverse cell signaling pathways of cancer cells. Clin Cancer Res 13: 1625–1629 17363512 10.1158/1078-0432.CCR-06-2966

[bib58] Young JC, Agashe VR, Siegers K, Hartl FU (2004) Pathways of chaperone-mediated protein folding in the cytosol. Nat Rev 5: 781–791 10.1038/nrm1492 15459659

[bib59] Zhang Y, Kwon S, Yamaguchi T, Cubizolles F, Rousseaux S, Kneissel M, Cao C, Li N, Cheng HL, Chua K, Lombard D, Mizeracki A, Matthias G, Alt FW, Khochbin S, Matthias P (2008) Mice lacking histone deacetylase 6 have hyperacetylated tubulin but are viable and develop normally. Mol Cell Biol 28: 1688–1701 18180281 10.1128/MCB.01154-06 PMC2258784

